# Real time measurement of myocardial substrate selection in vivo using hyperpolarized ^13^C magnetic resonance

**DOI:** 10.1186/1532-429X-17-S1-O15

**Published:** 2015-02-03

**Authors:** Jessica A Bastiaansen, Matthew E Merritt, Arnaud Comment

**Affiliations:** 1 Department of Radiology, University Hospital (CHUV) and University of Lausanne (UNIL), Lausanne, Switzerland; 2 Center for Biomedical Imaging (CIBM), Lausanne, Switzerland; 3 Advanced Imaging Research Center, Department of Radiology, University of Texas Southwestern Medical Center, Dallas, TX, USA; 4 Institute of Physics of Biological Systems, Ecole Polytechnique Fédérale de Lausanne, Lausanne, Switzerland

## Background

Cardiac dysfunction is often associated with a shift in substrate preference and metabolism, but current *in vivo* techniques only provide information on substrate uptake. Hyperpolarized (HP) MR has the unique ability to detect metabolism *in vivo* and is highly specific. To evaluate the prospects for measuring myocardial substrate selection *in vivo*, HP [1-^13^C]pyruvate and [1-^13^C]butyrate were co-infused into rats.

## Methods

Pyruvate and butyrate were hyperpolarized by DNP. After dissolution the samples were automatically infused into fed or fasted animals. Myocardial metabolism was detected using a train of cardiac triggered 30^o^ adiabatic inspection pulses applied every 3 s at 9.4T with ^1^H decoupling. Metabolite ratios were calculated to assess overall consumption of the imaging agents.

## Results

Downstream metabolites of butyrate including ketone bodies β-hydroxybutyrate (BHB) and acetoacetate, acetylcarnitine, butyrylcarnitine, citrate, and glutamate, were observed *in vivo* and affected by fasting (1A) and co-infusion of pyruvate (1B). Pyruvate competed with butyrate for the production of acetyl-CoA as evidenced by changes in the production of bicarbonate (2C), acetylcarnitine (1D,2E), glutamate (1E,1G) and acetoacetate (1C,1F).

### Fed state

Mitochondrial pseudoketogenesis facilitated the labeling of the ketone bodies, and no evidence of true ketogenesis was observed. The competition presented by butyrate results in a decrease in the bicarbonate signal (2C, 2G). From the perspective of butyrate metabolism, the glutamate (1E, 1G) decreased and the appearance of acetoacetate (1C, 1F) was nearly quenched. The acetylcarnitine increase (1D) and decrease in glutamate and acetoacetate signals were modulations due to competition from pyruvate for the limited number of free CoA units in the mitochondria, and the antiporting of acetoacetate when pyruvate enters the mitochondria.

### Fasted state

Different enzyme expressions and circulating substrates in the fasted myocardium reduced PDH flux (2C) and increased lactate, interpreted as a redox state increase. Glutamate was lower (1E, 1G) while pool sizes remain unchanged, indicating decreased ^13^C labeling. An increase in acetoacetate was observed (1C, 1F). With increased circulating ketones, label exchange into acetoacetate is facilitated, decreasing the fraction of label available for production of acetylCoA and glutamate. Acetylcarnitine (2E) rose and a drop in bicarbonate (2C, 2G) was observed. The change in glutamate to acetylcarnitine ratio decreased significantly upon introduction of pyruvate (1G).

## Conclusions

Pyruvate and butyrate competed for the production of acetyl-CoA and fasting produced logical changes in the observed spectra. Co-infusion of HP metabolic fuels is a one of a kind method for assessing myocardial substrate preference *in vivo*. The combination of HP ^13^C technology and co-administration of two separate imaging agents enables noninvasive and simultaneous monitoring of both fatty acid and carbohydrate oxidation in the heart*.*

## Funding

This work was supported by the Swiss National Science Foundation (grant PP00P2_133562 and 31003AB_131087), the Centre d'Imagerie BioMédicale (CIBM) of the UNIL, UNIGE, HUG, CHUV, EPFL, and the Leenaards and Jeantet Foundations. Matthew Merritt was supported by CPRIT RP-101243, NIH 5 R37 HL34557, NIH R21EB016197, and NIH-NIBIB 5 P41 EB015908.

**Figure 1:**
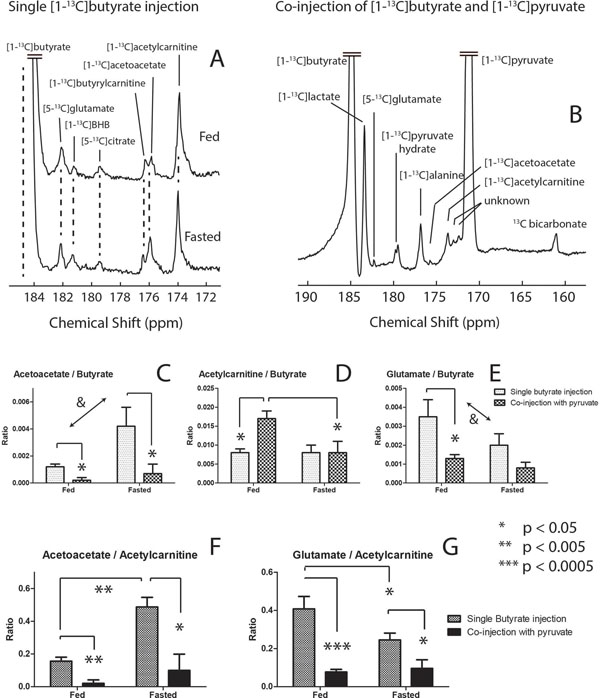
(A) *In vivo* cardiac ^13^C spectrum recorded after the injection and metabolism of hyperpolarized [1-^13^C]butyrate in the fed and fasted state (n=6 in both groups). (B) A sum spectrum with expansion to show the downstream metabolites of the co-injection experiments with both HP butyrate and pyruvate (n=7 in both groups). (C-E) The normalized signals relative to the injected substrate [1-^13^C]butyrate associated with metabolites of [1-^13^C]butyrate in the 4 experimental conditions. (F & G) Relative ratios of acetoacetate and glutamate to that of acetylcarnitine. The signals of glutamate and acetoacetate demonstrate the competition between pyruvate and butyrate for the production of acetyl-CoA as well as the impact of pyruvate on pseudoketogenesis. (&) Two way ANOVA reports a significant overall effect of the factor of nutritional state on the ratios of acetoacetate to butyrate and glutamate to butyrate. Error bars indicate +/- SEM.

**Figure 2:**
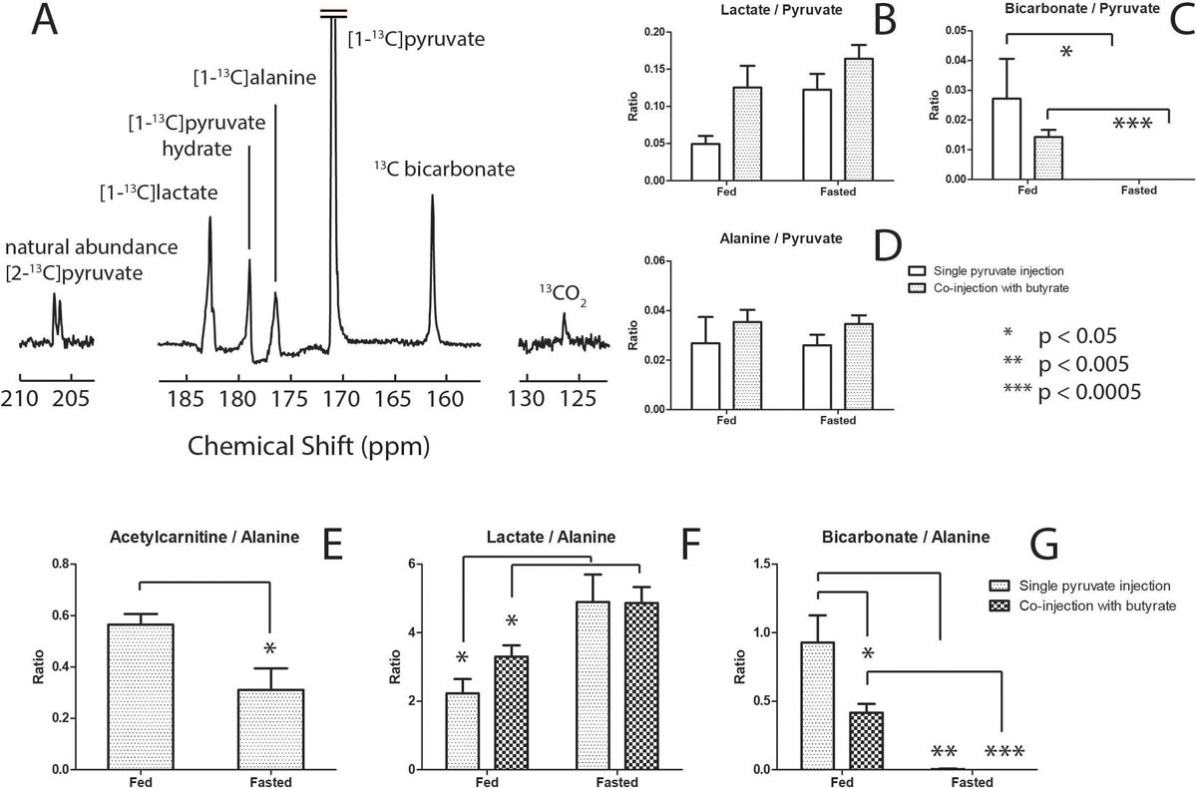
(A) *In vivo* cardiac ^13^C spectrum recorded after the injection and metabolism of hyperpolarized [1-^13^C]pyruvate in the fed state (n=5 in both groups). Signal ratios of lactate (B, F), bicarbonate (C,G) and acetylcarnitine (E) relative to pyruvate and alanine in all 4 experimental groups where hyperpolarized [1-^13^C]pyruvate was either injected separately or co-injected with hyperpolarized [1-^13^C]butyrate in both fed and fasted animals. Note the nearly constant fraction of alanine, which justifies its use as normalization factor for the other metabolites as was shown previously in perfused heart. Error bars indicate +/- SEM.

